# Exploratory comparison of PASC and SARS-CoV-2 infection through metabolomics and lipidomics in early pandemic and Omicron-era

**DOI:** 10.1016/j.bbrep.2026.102652

**Published:** 2026-05-30

**Authors:** Chelsea M. Rock, Laura M. Johnson, Adrianna L. Westbrook, Richard Parsons, John D. Roback, Gregory L. Damhorst, Wilbur A. Lam, Greg S. Martin, Madelyn C. Houser, Jennifer K. Frediani

**Affiliations:** a Department of Medicine, Emory University School of Medicine, Atlanta, GA, USA; b Pediatric Biostatistics Core, Department of Pediatrics, Emory University, Atlanta, GA, USA; c Nell Hodgson Woodruff School of Nursing, Emory University, Atlanta, GA, USA; d Department of Pathology and Laboratory Medicine, Emory University School of Medicine, Atlanta, GA, USA; e Division of Infectious Diseases, Department of Medicine, Emory University School of Medicine, Atlanta, GA, USA; f Department of Pediatrics, Emory University School of Medicine, Atlanta, GA, USA; g Aflac Cancer and Blood Disorders Center of Children's Healthcare of Atlanta, Atlanta, GA, USA; h Wallace H. Coulter Department of Biomedical Engineering, Emory University and Georgia Institute of Technology, Atlanta, GA, USA

**Keywords:** COVID-19, SARS-CoV-2, Metabolomics, Lipidomics, Plasma, Liquid chromatography-mass spectrometry, Hospitalization

## Abstract

While symptomology of post-acute sequelae of COVID-19 (PASC) is well-known, and more risk factors are being identified, the physiological mechanisms of this condition remain largely undetermined. In the early pandemic into the pre-Omicron era, plasma samples were obtained from SARS-CoV-2 positive patients admitted to the hospital or in the emergency department at a single timepoint. The 68 convenience samples were analyzed using metabolomics and lipidomics to identify any signatures associated with development of PASC. After correction for false discovery rate, no metabolomic features distinguished the 24% of patients that developed PASC from those who did not. Glycerophospholipids, including PI(20:4_18:0), and triglycerides, which are known to be involved in energy metabolism and immune responses, differed in abundance in PASC compared to non-PASC patients. A small sample size, batch effects, and inaccessible clinical data resulted in an exploratory analysis with moderate effects in this convenience sample. Discerning biological pathways in SARS-CoV-2 infection, PASC, and their linkage may help elucidate causes and uncover preventative and treatment approaches for patients with PASC.

## Highlights

• Lipid PI(20:4_18:0) downregulated in PASC, linked to inflammation and brain fog.
• 2022 cohort showed elevated hypoxanthine, urea, and l-glutamine levels.
• Ergocalciferol and 4-hydroxyquinoline reduced in Omicron-era infections.
• Lipidomic shifts suggest immune dysregulation may underlie PASC symptoms.

## 1 Introduction

Severe acute respiratory syndrome coronavirus 2 (SARS-CoV-2) infection can be a systemic illness, causing widespread inflammation and end-organ damage [1,2]. This physiology may lead to exacerbation of chronic diseases or contribute to development of post-viral conditions, such as the heterogeneous post-acute sequelae of COVID-19 (PASC) syndrome, commonly referred to as Long COVID, which is characterized by a wide range of symptoms lasting 1-3 months or longer after the acute illness [[3], [4], [5], [6], [7]]. PASC can commonly include fatigue and brain fog as well as cardiovascular [3,4,6,8] and renal [9,10] dysfunction. PASC has been documented in approximately 10 out of 100 unvaccinated individuals in the pre-Delta era, with prevalence decreasing somewhat as the Omicron variant became dominant and decreasing markedly for vaccinated individuals [11], though reports of prevalence range widely, and may be much higher (more than 80%) in patients hospitalized with COVID-19 [3,4]. While researchers have made some progress in unraveling the physiology of PASC and how it relates to the index SARS-CoV-2 infection, biological mechanisms remain largely undetermined [5].

Some evidence exists that disease severity may contribute to PASC [5,10]. Patients exhibiting 5 or more symptoms during the initial infection are at a greater risk of PASC [3]. In the early pandemic, gastrointestinal symptoms (e.g., nausea, loss of appetite) were more prevalent in PASC patients (30%) than in the population of acute SARS-CoV-2 patients (10%), which may suggest persistent SARS-CoV-2 infection or response to infection in non-respiratory organs and may be related to ACE2 receptor overexpression [4]. Patients who developed PASC following index infection in the Omicron-era reported fewer but similar symptoms compared to patients who had SARS-CoV-2 in early stages of the pandemic [12]. Elevated inflammatory markers have been found in PASC patients following initial infection [13]. Additionally, induction of a lipid mediator storm causing pro-inflammatory responses in COVID-19 patients [9] may contribute to multisystem inflammation resulting in chronic conditions such as PASC. Dysregulation of lipidomic and metabolomic pathways related to inflammation as well as protein degradation and energy metabolism have been found in those with PASC compared to controls [14]. In fact, a growing body of research is using untargeted and targeted approaches to examine metabolic and lipidomic signatures including possible biomarkers in SARS-CoV-2 [5,[15], [16], [17], [18], [19], [20], [21]]. Blood plasma is a primary carrier of metabolites, which makes it a suitable biological specimen for large analyses [19]. Using plasma in high-throughput methods to measure metabolites and lipids can provide clues to the physiological underpinnings of COVID-19 sequelae as they relate to multiple organ systems, including the cardiovascular, renal, nervous, respiratory, and immune systems [3,22], and can spur further targeted investigation.

As a preliminary investigation of the gap in knowledge of PASC risk factors and mechanisms, we analyzed metabolomic and lipidomic profiles in patients with SARS-CoV-2 infection presenting to the hospital in either 2020 (early pandemic) or 2022 (Omicron-era). This design allowed us to examine samples from the early pandemic when most patients were SARS-CoV-2 naïve, and from the Omicron-era, when both the circulating virus and the exposure landscape were somewhat altered. We sought to determine whether metabolomic or lipidomic features detectable in the acute infection stage could distinguish patients who would experience persistent symptoms from those who would recover more efficiently. We hypothesized that these features would include biomarkers related to affected bodily systems (e.g., cardiovascular, respiratory, hepatic, and gastrointestinal systems).

## 2 Methods

### 2.1 Study design and ethical approval

This observational study was reviewed and approved by the Emory University Institutional Review Board [IRB # STUDY00000510] for the use of residual specimens for analysis. A Health Insurance Portability and Accountability Act (HIPAA) authorization and consent waiver were granted by the IRB. Documentation of consent was not required.

### 2.2 Specimen collection and clinical data review

Clinical residual blood plasma specimens in EDTA from individuals presenting to the hospital who were positive for SARS-CoV-2 by nasopharyngeal polymerase chain reaction (PCR) were originally collected for a study of blood biomarkers in COVID-19 following standard of care assays on a hematology analyzer and stored at −20 °C (samples collected from 2020) or −80 °C (samples collected in 2022) until analysis. The inclusion criteria for the 2020 cohort included hospitalized adult patients with acute COVID-19 confirmed by nasopharyngeal PCR regardless of symptoms. We excluded patients who reported an objective COVID-19 diagnosis more than one week prior to admission or who had onset of COVID-like symptoms greater than 14 days prior to admission. The 2022 cohort included adult hospitalized patients that had a complete blood count collected within 12 h of the nasopharyngeal swab collection. Patients were excluded if the sample was not available or not viable.

Specimens and clinical data were available for 52 patients in 2020 and 54 patients in 2022 [23]. From this sampling, medical records were reviewed to determine whether the participants had been diagnosed with PASC. A participant was labeled as having PASC if an ICD-10 code had been recorded for PASC, the patient had been referred to a PASC subspecialty clinic, or if a clinician had documented symptoms compatible with PASC more than four weeks after their initial COVID-19 diagnosis [24]. The Long Covid definition used for this work was developed by the Department of Health and Human Services (HHS) in collaboration with CDC and other partners in 2022 [25]. Specimens were excluded from the analysis if PASC could not be determined, for example due to death during the index hospitalization or if they did not have follow-up within our healthcare system. A total of 76 patients were able to be classified, and their earliest collected plasma sample (i.e., within 24 h of admission) from the index hospitalization or emergency department encounter was assayed for broad metabolomic and lipidomic profiling. Data were reported for 68 viable samples. The analysis was hypothesis-generating, to identify any differences in metabolomic and lipidomic profiling between those who developed persistent symptoms following acute COVID-19 and those who did not.

### 2.3 Metabolomics and lipidomics methods

High resolution metabolomics was performed by the Emory Integrated Metabolomics and Lipidomics Core using previously published methods [26]. Briefly, metabolites were extracted from 50 μL plasma in 200 μL of a 1:1 mixture of acetonitrile:methanol (ACN:MeOH). Samples were analyzed using a multiple reaction monitoring-based LC-MS/MS method on an Agilent Infinity II 1290 LC system coupled with a 6495c mass spectrometer (Agilent, UK). The mass spectrometer was operated in both positive and negative ion modes using polarity switching. A 2 μL injection was analyzed on a HILIC-Z column using a 25-min gradient. Data were processed using Skyline software, with peak integration and relative concentration calculations performed using internal standards and an external calibration curve. Briefly, metabolites were extracted from 50 μL plasma in 200 μL of a 1:1 mixture of acetonitrile:methanol (ACN:MeOH). Samples were analyzed using a multiple reaction monitoring-based LC-MS/MS method on an Agilent Infinity II 1290 LC system coupled with a 6495c mass spectrometer (Agilent, UK). The mass spectrometer was operated in both positive and negative ion modes using polarity switching. A 2 μL injection was analyzed on a HILIC-Z column using a 25-min gradient [26]. Data were processed using Skyline software, with peak integration and relative concentration calculations performed using internal standards and an external calibration curve.

For lipidomic analysis, patient samples were processed as previously described in a cold room at 4 °C. Plasma samples (50 μL) were loaded into preconditioned wells with internal standards and mixed with methanol. High resolution lipidomics was performed as previously described [27]. For lipidomic analysis, patient samples were processed in a cold room at 4 °C. Plasma samples (50 μL) were loaded into preconditioned wells with internal standards and mixed with methanol. High resolution lipidomics was performed as previously described [27] on a Vanquish UHPLC system with a Thermo IDX Fusion mass spectrometer using a C18 column and a 15-min gradient (Thermo Scientific, Waltham, MA). A data-dependent acquisition method was used, with MS/MS spectra recorded at 30,000 full width at half maximum (FWHM) resolution. Lipid identification was conducted using LipidSearch 4.1.28 software (Thermo Fisher Scientific Inc., San Jose, CA, USA), with high-confidence identifications requiring specific fragmentation patterns.

### 2.4 Data post-processing and statistical analysis

Metabolites and lipids were excluded from analysis if they had ≥80% missing data. Missingness is summarized distributionally for all platforms (Supplemental Table 1); for metabolomics, we additionally report named metabolites with higher missingness to improve interpretability given the smaller feature set (Supplemental Table 2). For the remaining features, missing data were imputed with random forest techniques. All data were normalized by median and log-base-10 transformed. Fold change and t-tests were used to assess which features differed in abundance. No fold-change magnitude cutoff was applied (fold-change cutoff set to 1.0 in MetaboAnalyst), and fold change was calculated for descriptive purposes only due to the exploratory nature of this study. Due to multiple comparisons, a false discovery rate (FDR) adjustment was made to p-values using the Benjamini-Hochberg method, and the threshold for statistical significance was 0.05. Throughout the results, features meeting FDR-adjusted p < 0.05 are referred to as statistically significant, whereas features with unadjusted p < 0.05 are described as nominally associated and interpreted as exploratory. Random forest imputation was conducted with the missForest package in R (version 4.4.1) [28]. Volcano plots and violin plots were used to visually compare groups, and orthogonal partial least squares discriminant analysis (OPLS-DA) was employed to see which features contributed most to the differentiation, as indicated by compounds with the largest variable importance in projection score (VIP). Statistical analysis was performed in MetaboAnalyst 6.0 [29].

## 3 Results

Participants (n = 68) were 51% female and averaged 60 years of age (Table 1). They were split evenly between the cohort years (2020 and 2022) and overall had a 24% prevalence of PASC. Of those that developed PASC, 50% were female, and 56% were from the 2020 cohort. Overall, 93% required some level of oxygen supplementation during acute infection.

**Table 1:** Sample descriptives.

Variable	Overall	PASC		Cohort	
		No (N = 52)	Yes (N = 16)	2020 (N = 34)	2022 (N = 34)
	**Mean (SD)**	**Mean (SD)**	**Mean (SD)**	**Mean (SD)**	**Mean (SD)**
Age	60.0 (16.1)	58.8 (17.0)	64.0 (12.5)	63.4 (14.6)	56.6 (17.0)

	**N (%)**	**N (%)**	**N (%)**	**N (%)**	**N (%)**
Sex					
*Male*	33 (49%)	25 (48%)	8 (50%)	14 (41%)	19 (56%)
*Female*	35 (51%)	27 (52%)	8 (50%)	20 (59%)	15 (44%)
Cohort Year					
*2020*	34 (50%)	25 (48%)	9 (56%)	-	-
*2022*	34 (50%)	27 (52%)	7 (44%)	-	-
Post Acute Sequelae of SARS-CoV-2 (PASC)					
*No*	52 (76%)	-	-	25 (74%)	27 (79%)
*Yes*	16 (24%)	-	-	9 (26%)	7 (21%)
WHO Severity^a^					
*3*	5 (7.4%)	4 (8%)	1 (6%)	5 (15%)	0 (0%)
*4*	50 (74%)	41 (79%)	9 (56%)	24 (71%)	26 (76%)
*5*	10 (15%)	5 (10%)	5 (31%)	4 (12%)	6 (18%)
*6*	2 (2.9%)	1 (2%)	1 (6%)	1 (3%)	1 (3%)
*7*	1 (1.5%)	1 (2%)	0 (0%)	0 (0%)	1 (3%)

a WHO Severity defined as 3 = Room Air; 4 = Nasal Cannula; 5 = High-flow oxygen; 6 = Mechanical ventilation; 7 = Intubation.

### 3.1 Comparison of patients with and without PASC

**Fig. 1:**
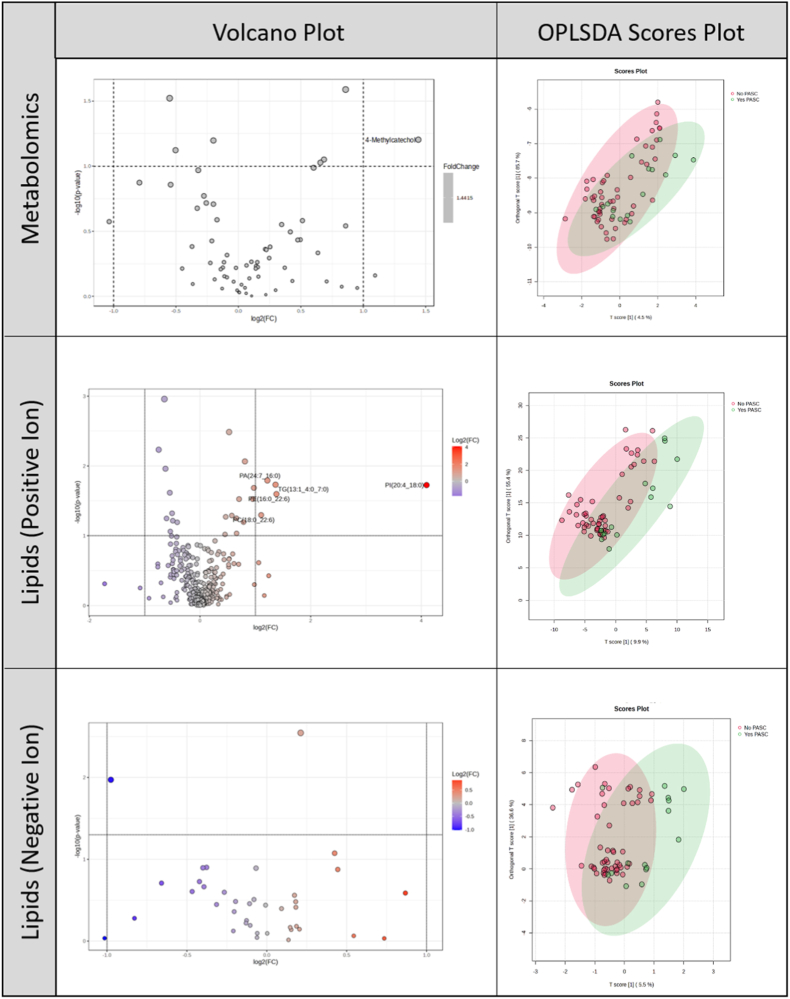
PASC analysis: Volcano plots and OPLS-DA scores plots for metabolomics and lipidomics. Legend: In volcano plots, fold change (FC) was calculated as non-PASC/PASC. Accordingly, FC > 1 (shown in red) indicates higher abundance in the non-PASC group, whereas FC < 1 (shown in blue) indicates higher abundance in the PASC group. In OPLS-DA plots, PASC=Yes is shown in blue and PASC=No is shown in red.

High-resolution metabolomics identified 66 total features but none that differed at FDR-adjusted p < 0.05 by *t*-test between those with PASC and those without (Supplemental Table 3, Fig. 1). Untargeted lipidomics identified 364 total features with 16 unique and named lipids which exhibited FDR-adjusted differences in abundance by *t*-test between PASC groups (Supplemental Table 4, Fig. 1). In data obtained using positive ionization mode, nine were statistically less abundant in those with PASC, and five were statistically more abundant in those with PASC. In negative ionization mode, one was statistically less abundant in those with PASC and one was statistically more abundant (Supplemental Table 4, Fig. 1). To isolate PASC-related differences in metabolomics and lipidomics profiles from differences related to other factors (cohort, viral variant, medications, etc.) we conducted OPLS-DA. The positive ion mode lipidomics analysis showed the greatest separation by OPLS-DA, driven by AcCa(18:1), VIP = 2.94; LPC(O-18:1), VIP = 2.42; and SM(d36:4), VIP = 2.41 among others, only one of which [AcCa(18:1)] was also identified by *t*-test.

To evaluate model robustness and potential overfitting in the context of high-dimensional data, performance metrics from OPLS-DA models were examined, including variance explained in X (R2X), variance explained in the outcome (R2Y), and cross-validated predictive ability (Q2). OPLS-DA models comparing participants with and without PASC showed limited predictive performance across metabolomics and lipidomics platforms (Supplemental Table 5). Predictive components explained a small proportion of total variance (R2X ≤ 10%), outcome variance was minimal (R2Y ≤ 0.11), and cross-validated Q2 values were near zero or negative, indicating poor generalizability.

### 3.2 Comparison of 2020 vs 2022 cohort

**Fig. 2:**
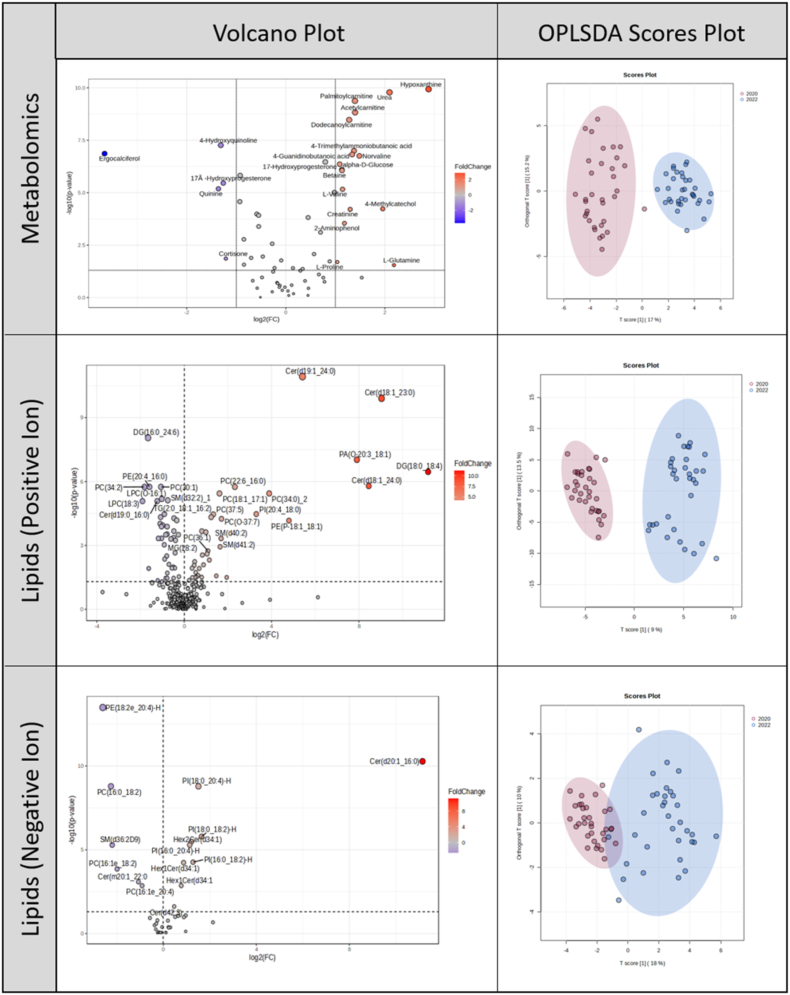
Cohort analysis: Volcano plots and OPLS-DA scores plots for metabolomics and lipidomics. Legend: In volcano plots, fold change (FC) was calculated as 2022/2020; FC > 1 (red) indicates higher abundance in 2022 and FC < 1 (blue) indicates higher abundance in 2020. In OPLS-DA plots, 2022 is shown in blue and 2020 in red.

Metabolomic and lipidomic profiles of the samples collected in 2020 and in 2022 may have differed for several reasons, including altered characteristics of the circulating SARS-CoV-2 viruses and the symptoms that they caused, altered patient responses to infection resulting from prior exposure and/or vaccination, and altered molecular composition of the samples due to different storage temperatures (−20 °C vs −80 °C). To aid in the interpretation of our comparison of profiles from patients who developed or did not develop PASC, we divided the samples by cohort year and identified 22 statistically significant, unique, and named metabolites more abundant and 15 less abundant in the 2022 cohort compared to the 2020 cohort (Supplemental Table 6, Fig. 2). These results were highlighted by full separation of metabolomic profiles by OPLS-DA (Fig. 2) driven by hypoxanthine (VIP = 1.70), urea (VIP = 1.70), palmitoylcarnitine (VIP = 1.66), and acetylcarnitine (VIP = 1.60), all of which were also identified by *t*-test.

The OPLS-DA for the PASC predictor indicated that the vast majority of the variation in the metabolomics data was explained by orthogonal factors (Fig. 1), of which cohort is a substantial contributor (Fig. 2). In keeping with this, we found that most of the metabolites nominally associated with PASC (Supplemental Table 3) also differed significantly in abundance by cohort (Supplemental Table 6). The PASC-associated metabolites which did not overlap with those identified in the cohort comparison were 1-methylnicotinamide, kynurenic acid, and l-isoleucine. These metabolites were prevalent in the samples, with relatively low missingness (Supplemental Table 2).

In the lipidomics cohort analysis, there were eight lipids statistically more abundant in the 2022 cohort in data acquired by positive ionization mode and seven statistically less abundant. In negative ionization mode data, there were nine lipids more abundant in the 2022 cohort and six less abundant (Supplemental Table 7, Fig. 2). Positive ionization mode data also exhibited separation by OPLS-DA, with the highest significance assigned to Cer(d19:1_24:0), VIP = 2.72 and Cer(d18:1_23:0), VIP = 2.67, both of which were also identified by *t*-test. Violin plots showing numerical density for high abundance compounds can be found in Supplemental Fig. 1. In contrast to the metabolomics data, factors other than PASC development explained less of the variation in the lipidomics data (Fig. 1), and we found that, with the exceptions of PC(34:2) and PC(16:1e_20:4), the lipids nominally or significantly associated with PASC (Supplemental Table 4) did not overlap with those differing significantly by cohort (Supplemental Table 7).

For cohort comparisons, OPLS-DA models demonstrated moderate to strong predictive performance, particularly in lipidomics data (Supplementary Table 8). Predictive components explained 10–25% of total variance (R2X) and accounted for substantial outcome variance (R2Y = 0.55–0.63), with robust cross-validated performance (Q2 = 0.51–0.61). These findings indicated a strong cohort-associated signal.

## 4 Discussion

This study utilized metabolomic and lipidomic profiling of residual blood plasma samples collected from patients hospitalized with SARS-CoV-2 infection in 2020 and 2022 to determine whether molecular markers in the acute phase of the disease could distinguish patients who would recover efficiently from those who would experience persistent symptoms or PASC. Twenty-four percent of the 68 patients profiled in this study met criteria for PASC. This is a somewhat higher percentage than that documented in some other reports [11], but well within the wide range found in the literature. PASC is defined differently across different studies, and this study utilized retrospective chart review at a relatively early timepoint post-infection (4 weeks) to classify participants.

We identified a few metabolites nominally associated (unadjusted p < 0.05) with PASC development, but none that were statistically significant after FDR correction, and nearly all of which also differed significantly by cohort (2020 vs 2022, with different freezer storage temperatures), suggesting that their correlation with PASC status was likely incidental. The only metabolites nominally associated with PASC which did not differ by cohort were 1-methylnicotinamide (1-MNA), kynurenic acid, and l-isoleucine, all of which were relatively more abundant in patients who would develop PASC.

1-MNA is a metabolite of NAD+, which is a critical cofactor in cellular energy production. NAD+ is often rapidly metabolized during acute infections, including SARS-CoV-2 infections, due in part to the energetic and proliferative demands of immune cells [30,31]. 1-MNA itself has anti-inflammatory properties and has actually been tested as a supplement to combat fatigue in patients recovering from COVID [32]. Involvement of the kynurenine metabolic pathway, which includes metabolites related to tryptophan [16,21], in acute COVID-19 has been documented, including indications of its activation in plasma, correlation with immunological and neurodegenerative markers in acute infection [33], and its association with cognitive impairment (e.g., brain fog) in PASC [8]. l-isoleucine is critical for SARS-CoV-2 replication, and its availability has been identified as the rate-limiting factor in viral shedding in infected individuals [34]. Plasma l-isoleucine levels have been positively correlated with risk of SARS-CoV-2 infection and risk of hospitalization with COVID-19 [35].

If increased abundance of these metabolites in acute infection in patients who later developed PASC were confirmed in larger studies, it would likely align with the hypothesis that more severe acute SARS-CoV-2 infection with heightened inflammatory responses increases risk for development of PASC. Corticosteroid therapy (e.g., dexamethasone), used to reduce hyperinflammatory responses in severe COVID-19, also increases tryptophan [21] and could increase kynurenic acid levels in plasma. Thus, differences in medication use, which were not available to us for analysis, could also be contributing to observed patterns in metabolomics data. This would still align with the previously stated hypothesis, as more severely ill patients would have been more likely to receive corticosteroid treatment. Tryptophan and isoleucine bioavailability in COVID-19 are also influenced by diet [34], and both, along with kynurenic acid, are known to be regulated by the gut microbiome; the involvement of these factors in PASC risk may be worth investigating in future studies.

Stronger associations with PASC development were found for lipids than for metabolites in this study. Some of the strongest and most common associations were with phosphatidylinositols (PIs), phosphatidylethanolamines (PEs), phosphatidylcholines (PCs), and triglycerides. Perturbation of glycerophospholipids has been consistently documented in COVID-19 in humans and animal models and has been associated with disease severity [36]. Glycerophospholipids and their derivatives such as lysophospholipids can serve as mediators of immune signaling. Similarly, circulating triglyceride levels typically increase during acute infections, and they have been correlated with COVID-19 severity [9,17]. Lipidomics studies increasingly point to a lipid mediator storm increasing fatty acid circulation in tissue of PASC patients [9,17]. At hospital admission, we observed lower levels of PI(20:4_18:0), PE(16:0_22:6), and two triglyceride species in patients who would develop PASC and higher levels of LPC(18:2). Several PC species were more abundant and several species less abundant in the PASC group. Numerous studies have documented persistent alterations in these same subclasses of lipids in PASC patients [36,37]. It has also been reported that some of these lipids were elevated relative to controls during acute COVID-19 infection and then decreased relative to controls in PASC patients [14]. These persistent alterations in lipid profiles likely reflect altered energy metabolism and inflammatory processes in PASC, although inconsistent findings across studies with regard to specific lipid species and to their direction of change complicate interpretation and linkages to physiological mechanisms. Interestingly, the lipid with the greatest reduction in patients that developed PASC relative to those who did not in our study, PI(20:4_18:0), a plasmalogen that plays a role in oxidative stress and inflammation, is related to neurodegenerative diseases. Degradation of PIs has been discussed as a pathway involved in PASC-related symptoms of fatigue or “brain fog,” analogous to its role in neurodegenerative and age-related diseases in human and animal models [38].

Overall, OPLS-DA indicated that only a small percentage of variance in the data could be linked to PASC development, and the model was poorly predictive. This could suggest that metabolic and lipidomic alterations which contribute to persistent COVID-19 sequelae had not yet manifested at the stage of acute illness we examined; sample collection at hospital admission could have been too early in the disease trajectory to detect differences indicative of PASC development. Additionally, though, there were structural limitations inherent in this study which constrained our ability to identify all PASC-related patterns which could have been present. Our exploratory study utilized remnant clinical specimens, with variation introduced by uncontrolled variables such as sex, age, circadian rhythm, exercise, drugs, diet, and other medical conditions [[39], [40], [41]]. These genetic and environmental variables influence pathophysiological states at the individual level [[39], [40], [41]]. For instance, information on corticosteroid or other treatment regimens, which have known effects on circulating metabolites [42], vaccination status, or kidney function was not accessible; these and other factors could significantly influence metabolomic and lipidomic profiles. Concordantly, OPLS-DA indicated that a large proportion of total variance in the data was captured by orthogonal components, indicating substantial structural variation unrelated to the outcome of interest.

Additionally, preanalytical factors, such as procedures for sample collection, handling, and storage, impact results [40]; these could not be controlled for our study despite specimens being processed and stored according to clinical laboratory protocols prior to use for research. These challenges were compounded by the integration of two cohorts of samples, one from 2020 and one from 2022. Utilization of these batches of samples broadened the generalizability of the PASC findings by incorporating data from a different stage of the pandemic, with potential differences in viral vectors, patient exposure and/or vaccination status, and treatments. This also necessarily increased variation in the samples, however, particularly because clinical laboratory protocols for storage shifted in the interim, and samples from the two cohorts were stored at different temperatures. 2020 cohort samples were stored at −20 °C while 2022 cohort samples were stored at −80 °C. While some molecules remain relatively stable in isolated plasma, significant alteration of metabolite composition over time has been documented in samples stored at −20 °C [43]. Our study found robust differences in metabolite and lipid profiles between the two cohorts. Some of these differences may be effects of different SARS-CoV-2 variants or patient responses to infection at different stages of the pandemic, but they could also be attributable to differential rates of degradation and enzymatic activity at different storage temperatures.

Acknowledging this potential confounder, we compared metabolomic and lipidomic features differing in abundance with PASC development classification to those differing in abundance between cohorts, reasoning that features associated with PASC which also differed significantly between cohorts may be less reliable due to the batch effect. There was substantial overlap in metabolites identified in the two comparisons, but little overlap in lipids. While lipids can be vulnerable to modification and degradation, studies have found that some types of lipids, such as cholesterol, triglycerides, and various hormones, remain relatively stable with even long-term storage at approximately −20 °C [44,45]. Other lipid subclasses such as free fatty acids [44], which were not measured in this study, are much less stable.

Other limitations in this study include variation in SARS-CoV-2 infection duration, though we attempted to constrain this range with exclusion criteria, and a small sample size, which limited power for both feature-level inference and pathway enrichment, underscoring the need for larger, cohort-balanced studies to support reliable pathway-level interpretation. Furthermore, while internal references were used to identify specific features, additional biochemical validation was not conducted. Our findings should be interpreted as exploratory and hypothesis-generating.

## 5 Conclusion

Our work contributes to the expanding study of metabolomics and lipidomics in SARS-CoV-2 and to an even smaller subset of studies including hospitalized patients (approximately 40 studies to date). These studies largely identify biomarkers [46], changes in phenotypes [47], and pathogenesis [48]. Our efforts to identify molecular indicators in acute infection related to the development of PASC revealed that PASC-associated differences at this stage of disease were quite modest, but also highlighted several lipid classes and species known to be perturbed in both acute COVID-19 and in PASC which may be worthy of further study in the search for biomarkers, risk factors, and treatment strategies for this serious condition. As time distances us from the start of the pandemic, more of this type of work should focus on metabolic pathways to understand the biological processes involved in the resolution of SARS-CoV-2 infection and the development of PASC.

## Resource availability

**Resource availability:** https://github.com/rbparso/Omics-Cohort-Paper-Datasets.

## Author contributions

Study design and conceptualization: J.K.F., W.A.L., G.S.M. Specimen collection: J.R. Statistical analysis: L.J., R.P., A.W., M.C.H. Writing of the original draft of the manuscript: C.R., J.K.F., G.D., Interpretation of results: C.R., J.K.F., M.C.H. Manuscript review/editing: C.R., J.K.F., G.D., L.J., M.C.H. All the authors reviewed and approved the manuscript.

## Declaration of competing interest

The authors declare the following financial interests/personal relationships which may be considered as potential competing interests:Wilbur Lam reports financial support was provided by Emory University School of Medicine. If there are other authors, they declare that they have no known competing financial interests or personal relationships that could have appeared to influence the work reported in this paper.

## Data Availability

Data will be made available on request.
