# Supplementary material for: Exploratory comparison of PASC and SARS-CoV-2 infection through metabolomics and lipidomics in early pandemic and Omicron-era
Source: Biochem Biophys Rep. 2026 May 30;46:102652. doi: 10.1016/j.bbrep.2026.102652 (PMC13241759; doi:10.1016/j.bbrep.2026.102652)
Supplement: Multimedia component 1 [file mmc1.docx]

**Supplemental Materials**

Supplemental Table 1. Summary of Feature Missingness

| **Missingness range** | **Metabolomics (n, %)** | **Lipidomics + (n, %)** | **Lipidomics – (n, %)** |
| --- | --- | --- | --- |
| 0–9% | 0 (0%) | 302 (93.5%) | 22 (54.7%) |
| 10–29% | 16 (9.8%) | 16 (5.0%) | 16 (39.0%) |
| 30–49% | 23 (14.0%) | 4 (1.2%) | 3 (7.3%) |
| 50–79% | 27 (16.5%) | 1 (0.3%) | 0 (%) |
| 80-99% (excluded) | 11 (6.7%) | 0 (0%) | 0 (%) |
| 100% (excluded) | 87 (53.0%) | 0 (0%) | 0 (%) |
| Total | 164 (100%) | 323 (100%) | 41 (100%) |

Supplemental Table 2. Missingness for each Metabolite Included in the Analysis

| % Missing Category | Metabolite | % Missing |  | Metabolite | % Missing |
| --- | --- | --- | --- | --- | --- |
| 70-79% | L-Tyrosine | 79% |  | 2-Methylglutaric Acid | 77% |
|  | Nicotinic acid | 79% |  | Adenosine | 73% |
|  | L-Nicotine | 78% |  | Dihydrouracil | 71% |
|  | Citrulline | 77% |  | N-Acetylaspartate | 71% |
|  | Methyl-B-D-galactopyranoside | 77% |  |  |  |
| 60-69% | Palmitoylcarnitine | 68% |  | Fructose | 65% |
|  | Dodecanoylcarnitine | 68% |  | L-Tryptophan | 64% |
|  | Biliverdin-IX | 67% |  | Acetylcarnitine | 62% |
|  | Pyrocatechol | 65% |  | Thymidine | 62% |
|  | Epicatechin | 65% |  |  |  |
| 50-59% | 3-Methyl-L-Histidine | 59% |  | Phosphocholine | 54% |
|  | Adonitol | 59% |  | L-Phenylalanine | 53% |
|  | Glutaryl-L-carnitine | 59% |  | Glucosamine 6-Sulfate | 52% |
|  | Uridine | 59% |  | 3-Carboxypropyl trimethylammonium | 50% |
|  | (ÃƒÂ±)-3-Hydroxybutyric acid | 57% |  |  |  |
| 40-49% | 3-Amino-4-Hydroxybenzoic Acid | 47% |  | Theobromine | 43% |
|  | 3-Hydroxy-2-Methylpyridine | 46% |  | 3-Methyl-2-buten-1-ol | 42% |
|  | Theophylline | 45% |  | 3R-hydroxy-isobutyric acid | 42% |
|  | Vanillin | 44% |  | Quinine | 41% |
|  | 1,7-Dimethylxanthine | 43% |  | ÃƒÂ -D-Glucose | 40% |
|  | 17ÃƒÂ -Hydroxyprogesterone | 43% |  |  |  |
| 30-39% | 4-Guanidobutyric acid | 38% |  | Nicotinamide | 33% |
|  | L-Norvaline | 38% |  | 1-Methylnicotinamide | 33% |
|  | L-Isoleucine | 36% |  | Purine | 32% |
|  | 5-Deoxy-5-(methylthio)adenosine | 36% |  | Estrone | 32% |
|  | L-Leucine | 35% |  | Trigonelline | 31% |
|  | Kynurenic acid | 34% |  | L-Proline | 30% |
| 20-29% | Creatinine | 29% |  | 2-Aminophenol | 23% |
|  | Caffeine | 29% |  | Betaine | 22% |
|  | L-Glutamine | 28% |  | L-Valine | 21% |
|  | Hypoxanthine | 24% |  | Glycerophosphocholine | 21% |
|  | Norleucine | 24% |  | 4-Quinolinol | 20% |
| <20% | Corticosterone | 18% |  | 4-Methylcatechol | 15% |
|  | Urea | 18% |  | Cortisone | 15% |
|  | 17-Hydroxyprogesterone | 17% |  | Vitamin D2 (Ergocalciferol) | 15% |

**Supplemental Table 3. PASC Analysis: Metabolomics Volcano Plot Results**

|  | Ion Mode | Retention Time | *m/z* Ratio* | FC^†^ | log2(FC) | p-value^‡^ | -LOG10(p) |
| --- | --- | --- | --- | --- | --- | --- | --- |
| **Metabolites in Less Abundance among PASC=Yes** | | | | | | | |
| 4-Methylcatechol | - | 0.76 | 108 | 2.72 | 1.44 | 0.79 | 0.10 |
| Cortisone | + | 1.52 | 91.1 | 2.14 | 1.10 | 0.96 | 0.02 |
| 1-Methylnicotinamide | + | 7.58 | 94.1 | 1.93 | 0.95 | 0.97 | 0.01 |
| 2-Aminophenol | - | 0.79 | 107.1 | 1.81 | 0.86 | 0.93 | 0.03 |
| L-Proline | + | 7.52 | 70.2 | 1.81 | 0.86 | 0.79 | 0.10 |
| Kynurenic acid | + | 2.49 | 89.1 | 1.78 | 0.83 | 0.97 | 0.01 |
| Creatinine | + | 2.89 | 44.2 | 1.63 | 0.71 | 0.96 | 0.02 |
| 4-Guanidinobutanoic acid | + | 10.27 | 87.1 | 1.61 | 0.69 | 0.79 | 0.10 |
| L-Isoleucine | + | 5.64 | 86.1 | 1.58 | 0.66 | 0.79 | 0.10 |
| Urea | + | 2.10 | 44.1 | 1.55 | 0.64 | 0.93 | 0.03 |
| 4-Trimethylammoniobutanoic acid* | + | 10.27 | 45.2  87.1 | 1.52 | 0.60 | 0.79 | 0.10 |
| **Metabolites in High Abundance among PASC=Yes** | | | | | | | |
| Epicatechin | + | 0.88 | 77.1 | 0.49 | -1.04 | 0.93 | 0.03 |
| Ergocalciferol | + | 0.87 | 379.3 | 0.58 | -0.79 | 0.83 | 0.08 |
| *Duplicate compounds; the m/z are not unique per species.  ^†^ Fold change (FC) was calculated as non-PASC / PASC; FC > 1 indicates higher abundance in the non-PASC group and FC < 1 indicates higher abundance in the PASC group.  ^‡^ p-value has been corrected using the false discovery rate method (Benjamini-Hochberg) | | | | | | | |

**Supplemental Table 4. PASC Analysis: Lipidomics Volcano Plot Results**

| Lipid | FC^†^ | log2(FC) | p-value^‡^ | -LOG10(p) | |
| --- | --- | --- | --- | --- | --- |
| **Positive Ion Channel** | | | | | |
| ***Lipids in Less Abundance among PASC=Yes*** | | | |  |  |
| PI(20:4_18:0) | 17.09 | 4.09 | **0.019** | **1.73** |  |
| PE(16:0_22:6) | 2.60 | 1.38 | **0.025** | **1.60** |  |
| TG(13:1_4:0_7:0) | 2.58 | 1.37 | **0.018** | **1.73** |  |
| PA(24:7_16:0) | 2.32 | 1.22 | **0.016** | **1.79** |  |
| PC(18:0_22:6) | 2.15 | 1.10 | 0.051 | 1.30 |  |
| AcCa(20:4) | 1.96 | 0.97 | **0.021** | **1.69** |  |
| PC(35:6) | 1.94 | 0.95 | **0.030** | **1.53** |  |
| TG(2:0_18:1_16:2) | 1.76 | 0.81 | **0.009** | **2.07** |  |
| PC(20:3_18:0)_1 | 1.73 | 0.79 | 0.064 | 1.19 |  |
| PC(16:0_16:1) | 1.63 | 0.71 | **0.030** | **1.53** |  |
| PC(O-39:9)_2 | 1.58 | 0.66 | 0.093 | 1.03 |  |
| LPC(22:4) | 1.58 | 0.66 | 0.056 | 1.25 |  |
| Cer(d20:1_17:1) | 1.49 | 0.57 | 0.051 | 1.29 |  |
| AcCa(18:1) | 1.44 | 0.53 | **0.003** | **2.49** |  |
| PC(16:1_16:0) | 1.38 | 0.46 | 0.054 | 1.27 |  |
| PC(35:3) | 1.37 | 0.45 | 0.096 | 1.02 |  |
| ***Lipids in High Abundance among PASC=Yes*** | | | |  |  |
| LPC(18:2) | 0.60 | -0.75 | **0.006** | **2.23** |  |
| PC(O-39:10)_1 | 0.64 | -0.64 | **0.001** | **2.96** |  |
| SM(d44:5) | 0.65 | -0.62 | **0.011** | **1.96** |  |
| SM(d36:4) | 0.66 | -0.61 | 0.057 | 1.25 |  |
| PC(O-39:10) | 0.68 | -0.55 | **0.024** | **1.62** |  |
| PC(34:2) | 0.69 | -0.54 | 0.087 | 1.06 |  |
| PC(O-39:8)_2 | 0.70 | -0.50 | **0.048** | **1.32** |  |
| SM(d42:4) | 0.71 | -0.49 | 0.061 | 1.21 |  |
| PC(P-39:10) | 0.75 | -0.41 | 0.064 | 1.19 |  |
| **Negative Ion Channel** | | | |  |  |
| ***Lipids in Less Abundance among PASC=Yes*** | | | |  |  |
| PC(8:0e_10:0) | 1.34 | 0.42 | 0.084 | 1.07 |  |
| PC(16:1e_20:4) | 1.16 | 0.21 | **0.003** | **2.54** |  |
| ***Lipids in High Abundance among PASC=Yes*** | | | |  |  |
| PE(16:1e_20:4)-H | 0.51 | -0.98 | **0.011** | **1.97** |  |
| ^†^ Fold change (FC) was calculated as non-PASC / PASC; FC > 1 indicates higher abundance in the non-PASC group and FC < 1 indicates higher abundance in the PASC group.  ^‡^ p-value has been corrected using the false discovery rate method (Benjamini-Hochberg) | | | | |  |

Supplemental Table 5.

|  |  | **Predictive (p1)** | | | **Orthogonal (o1)** | | |
| --- | --- | --- | --- | --- | --- | --- | --- |
| **Outcome** | **Analysis** | **R2X** | **R2Y** | **Q2** | **R2X** | **R2Y** | **Q2** |
| **PASC** | **Metabolomics** | **0.05** | **0.06** | **-0.04** | **0.86** | **0.04** | **0.01** |
| **PASC** | **Lipidomics (+)** | **0.10** | **0.11** | **-0.06** | **0.55** | **0.08** | **-0.02** |
| **PASC** | **Lipidomics (-)** | **0.06** | **0.11** | **0.01** | **0.37** | **0.22** | **0.02** |

**Supplemental Table 6. Cohort Analysis: Metabolomics Volcano Plot Results**

| Metabolite |  | Ion  Mode | Retention Time | *m/z* Ratio* | FC^†^ | log2(FC) | p-value^‡^ | -LOG10(p) |
| --- | --- | --- | --- | --- | --- | --- | --- | --- |
| **Metabolites in High Abundance in 2022** | | | | | | | | |
| Hypoxanthine |  | + | 2.96 | 119 | 7.38 | 2.88 | **5.43E-09** | **8.27** |
| L-Glutamine |  | + | 9.99 | 84.1 | 4.54 | 2.18 | 0.05 | 1.32 |
| Urea |  | + | 2.10 | 44.1 | 4.27 | 2.09 | **5.43E-09** | **8.27** |
| 4-Methylcatechol |  | - | 0.76 | 108 | 3.88 | 1.96 | **0** | **3.73** |
| Norvaline |  | + | 6.32 | 42.2 | 2.8 | 1.49 | **1.16E-06** | **5.93** |
| Palmitoylcarnitine |  | + | 2.4 | 85.1 | 2.63 | 1.4 | **9.25E-09** | **8.03** |
| Acetylcarnitine |  | + | 7.76 | 85.1 | 2.64 | 1.4 | **2.46E-08** | **7.61** |
| 4-Trimethylammoniobutanoic acid* | | + | 10.27 | 45.2  87.1 | 2.60 | 1.38 | **9.48E-07** | **6.02** |
| 4-Guanidinobutanoic acid |  | + | 10.27 | 87.1 | 2.53 | 1.34 | **1.11E-06** | **5.96** |
| Creatinine |  | + | 2.89 | 44.2 | 2.46 | 1.3 | **0** | **3.73** |
| Dodecanoylcarnitine |  | + | 2.94 | 85.1 | 2.43 | 1.28 | **4.45E-08** | **7.35** |
| 2-Aminophenol* |  | - | 7.58  0.79 | 94.1  107.1 | 2.27 | 1.18 | **0** | **3.14** |
| L-Valine |  | - | 6.4 | 42.2 | 2.21 | 1.15 | **2.53E-05** | **4.60** |
| Betaine |  | + | 6.18 | 59.2 | 2.2 | 1.14 | **4.12E-06** | **5.38** |
| alpha-D-Glucose* |  | - | 6.4 | 59.2  89.0 | 2.19 | 1.13 | **4.03E-06** | **5.39** |
| 17-Hydroxyprogesterone* |  | + | 0.98 | 285.2  301.2 | 2.13 | 1.09 | **2.41E-06** | **5.62** |
| L-Proline |  | + | 7.52 | 70.2 | 2.06 | 1.04 | **0.04** | **1.44** |
| 3R-hydroxy-isobutyric acid* | | + | 3.47 | 45.2  57.2 | 1.97 | 0.98 | **3.38E-05** | **4.47** |
| L-Phenylalanine | | + | 5.22 | 120.1 | 1.85 | 0.89 | **0.03** | **1.46** |
| 3-Methyl-2-buten-1-ol | | + | 6.87 | 60.2 | 1.74 | 0.8 | **2.05E-06** | **5.69** |
| 5-Deoxy-5-(methylthio)adenosine | | + | 1.30 | 136 | 1.63 | 0.7 | **0** | **2.74** |
| Adenosine | | + | 2.43 | 119 | 1.49 | 0.57 | **0.02** | **1.74** |
| Adonitol |  | - | 5.14 | 89.1 | 1.37 | 0.45 | **0** | **3.39** |
| Corticosterone | | + | 0.78 | 91.1 | 1.27 | 0.34 | 0.07 | 1.17 |
| Estrone |  | + | 0.80 | 77.1 | 1.23 | 0.29 | 0.08 | 1.10 |
| Trigonelline | | + | 6.53 | 92.1 | 1.11 | 0.14 | 0.06 | 1.20 |
| **Metabolites in High Abundance in 2020** | | | | | | | | |
| Ergocalciferol |  | + | 0.87 | 379.3 | 0.08 | -3.66 | **1.11E-06** | **5.96** |
| Quinine |  | + | 1.20 | 81.1 | 0.39 | -1.36 | **2.53E-05** | **4.6** |
| 4-Hydroxyquinoline |  | + | 1.12 | 51.2 | 0.40 | -1.31 | **6.01E-07** | **6.22** |
| 17Ã-Hydroxyprogesterone* |  | - | 0.98 | 301.2  285.2 | 0.42 | -1.26 | **1.44E-05** | **4.84** |
| Cortisone |  | + | 1.52 | 91.1 | 0.43 | -1.21 | **0.03** | **1.57** |
| Epicatechin | | + | 0.88 | 77.1 | 0.53 | -0.93 | **8.78E-05** | **4.06** |
| Dihydrouracil | | + | 1.05 | 44.3 | 0.53 | -0.92 | **6.64E-06** | **5.18** |
| Fructose* |  | - | 4.79 | 59.2  89.1 | 0.56 | -0.85 | **0.00** | **2.42** |
| 1,7-Dimethylxanthine | | + | 1.18 | 124 | 0.56 | -0.84 | **0.05** | **1.33** |
| Theobromine | | + | 1.15 | 138 | 0.62 | -0.7 | **0.03** | **1.59** |
| Purine |  | + | 0.85 | 67.1 | 0.67 | -0.59 | **0.00** | **3.53** |
| Phosphocholine | | + | 12.13 | 125 | 0.68 | -0.55 | **0** | **3.48** |
| L-Tryptophan | | + | 5.88 | 188 | 0.68 | -0.55 | **0** | **3.00** |
| Vanillin |  | + | 1.05 | 53.2 | 0.72 | -0.46 | **0.02** | **1.64** |
| Pyrocatechol | | + | 1.15 | 39.2 | 0.83 | -0.27 | **0.01** | **2.22** |
| N-Acetylaspartate* | | + | 10.71 | 43.2  134 | 0.84 | -0.25 | 0.09 | 1.06 |
| *Duplicate compounds; the m/z are not unique per species.  ^†^ Fold change (FC) was calculated as the ratio of mean abundance in the 2022 cohort divided by the mean abundance in the 2020 cohort (2022 / 2020); FC > 1 indicates higher abundance in the 2022 cohort and FC < 1 indicates higher abundance in the 2020 cohort.  ^‡^ p-value has been corrected using the false discovery rate method (Benjamini-Hochberg) | | | | | | | | |

**Supplemental Table 7. Cohort Analysis: Lipidomics Volcano Plot Results**

| Lipid | FC^†^ | log2(FC) | p-value^‡^ | -LOG10(p) |
| --- | --- | --- | --- | --- |
| **Positive Ion Channel** | | | | |
| ***Lipids in Higher Abundance in 2022*** | | | | |
| DG(18:0_18:4) | 2300.4 | 11.17 | **6.15E-09** | **8.21** |
| Cer(d18:1_23:0) | 524.84 | 9.04 | **8.76E-13** | **12.06** |
| Cer(d18:1_24:0) | 349.33 | 8.45 | **3.42E-08** | **7.47** |
| PA(O-20:3_18:1) | 239.82 | 7.91 | **1.33E-09** | **8.88** |
| Cer(d19:1_24:0) | 42.73 | 5.42 | **4.15E-14** | **13.38** |
| PC(34:0)_2 | 14.85 | 3.89 | **1.53E-07** | **6.82** |
| PC(22:6_16:0) | 5 | 2.32 | **5.15E-08** | **7.29** |
| PC(18:1_17:1) | 3.06 | 1.61 | **1.44E-07** | **6.84** |
| ***Lipids in Higher Abundance in 2020*** | | | | |
| LPC(18:3) | 0.27 | -1.91 | **4.45E-07** | **6.35** |
| PC(34:2) | 0.28 | -1.82 | **6.28E-08** | **7.2** |
| DG(16:0_24:6) | 0.32 | -1.65 | **9.35E-11** | **10.03** |
| PE(20:4_16:0) | 0.33 | -1.59 | **5.71E-08** | **7.24** |
| PC(30:1) | 0.48 | -1.06 | **5.88E-08** | **7.23** |
| LPC(O-16:1) | 0.49 | -1.03 | **3.02E-07** | **6.52** |
| SM(d32:2)_1 | 0.59 | -0.75 | **3.74E-07** | **6.43** |
| **Negative Ion Channel** | | | | |
| ***Lipids in Higher Abundance in 2022*** | | | | |
| Cer(d20:1_16:0) | 2270.10 | 11.15 | **2.69E-12** | **11.57** |
| PI(18:0_18:2)-H | 3.12 | 1.64 | **2.04E-07** | **6.69** |
| PI(18:0_20:4)-H | 2.84 | 1.51 | **1.45E-10** | **9.84** |
| PI(16:0_18:2)-H | 2.42 | 1.28 | **1.26E-05** | **4.9** |
| Hex2Cer(d34:1) | 2.31 | 1.21 | **4.94E-07** | **6.31** |
| PI(16:0_20:4)-H | 2.19 | 1.13 | **1.06E-06** | **5.98** |
| Hex1Cer(d34:1) | 1.83 | 0.87 | **1.49E-05** | **4.83** |
| Hex1Cer(d34:1 | 1.70 | 0.77 | **0.00049** | **3.31** |
| Cer(d42:3) | 1.38 | 0.46 | **0.009309** | **2.03** |
| ***Lipids in Higher Abundance in 2020*** | | | | |
| PE(18:2e_20:4)-H | 0.16 | -2.61 | **8.19E-16** | **15.09** |
| PC(16:0_18:2) | 0.21 | -2.26 | **1.66E-10** | **9.78** |
| SM(d36:2D9) | 0.22 | -2.20 | **9.84E-07** | **6.01** |
| PC(16:1e_18:2) | 0.25 | -1.99 | **3.94E-05** | **4.40** |
| Cer(m20:1_22:0 | 0.47 | -1.08 | **0.000252** | **3.60** |
| PC(16:1e_20:4) | 0.53 | -0.91 | **0.000495** | **3.31** |
| ^†^ FC values > 1 indicate elevated levels in 2022 and FC values < 1 indicate elevated levels in 2020  ^‡^ p-value has been corrected using the false discovery rate method (Benjamini-Hochberg) | | | | |

**Supplemental Figure 1. Violin Plots (Metabolomics and Lipidomics)**


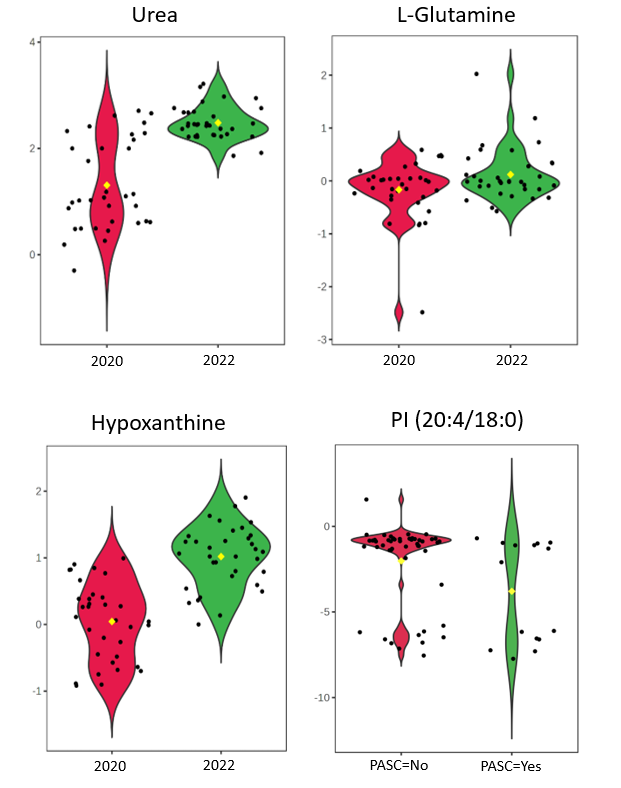


Supplemental Table 8.

|  |  | **Predictive (p1)** | | | **Orthogonal (o1)** | | |
| --- | --- | --- | --- | --- | --- | --- | --- |
| **Outcome** | **Analysis** | **R2X** | **R2Y** | **Q2** | **R2X** | **R2Y** | **Q2** |
| **Cohort** | **Metabolomics** | **0.03** | **0.23** | **0.22** | **0.85** | **0.35** | **0.32** |
| **Cohort** | **Lipidomics (+)** | **0.10** | **0.55** | **0.51** | **0.56** | **0.08** | **0.07** |
| **Cohort** | **Lipidomics (-)** | **0.25** | **0.63** | **0.61** | **0.38** | **0.02** | **0.01** |
